# Cardiorespiratory fitness and cardiovascular risk among in-patients with depression compared to healthy controls

**DOI:** 10.3389/fpsyt.2023.1193004

**Published:** 2023-06-20

**Authors:** Markus Gerber, Robyn Cody, Johannes Beck, Serge Brand, Lars Donath, Anne Eckert, Martin Hatzinger, Christian Imboden, Jan-Niklas Kreppke, Undine E. Lang, Sebastian Ludyga, Sarah Mans, Thorsten Mikoteit, Anja Oswald, Nina Schweinfurth-Keck, Lukas Zahner, Oliver Faude

**Affiliations:** ^1^Department for Sport, Exercise and Health, University of Basel, Basel, Switzerland; ^2^Psychiatric Clinic Sonnenhalde, Riehen, Switzerland; ^3^Adult Psychiatric Clinics (UPKE), University of Basel, Basel, Switzerland; ^4^Sleep Disorders Research Center, Kermanshah University of Medical Sciences, Kermanshah, Iran; ^5^Substance Use Prevention Research Center and Sleep Disorder Research Center, Kermanshah, University of Medical Sciences (KUMS), Kermanshah, Iran; ^6^School of Medicine, Tehran University of Medical Sciences (TUMS), Tehran, Iran; ^7^German Sport University Cologne, Cologne, Germany; ^8^Psychiatric Services Solothurn, Solothurn, Switzerland, and University of Basel, Solothurn, Switzerland; ^9^Private Clinic Wyss, Münchenbuchsee, Switzerland

**Keywords:** VO_2_max, overweight, major depression, blood pressure, cholesterol, triglycerides, HbA1c

## Abstract

**Introduction:**

Compared to the general population, individuals with depression have an increased risk for cardiovascular diseases. Nevertheless, little is known so far whether cardiorespiratory fitness (CRF) moderates this relationship. Therefore, we examined whether common physiological cardiovascular risk factors differ between patients with depression and healthy (non-depressed) controls, whether patients and controls differ in CRF, and whether higher CRF is associated with a lower cardiovascular risk in both patients and healthy controls. Additionally, we examined whether within the patient sample, cardiovascular risk factors differ between patients with mild, moderate and severe depression, and whether the relationship between symptom severity and cardiovascular risk is moderated by patients’ CRF levels.

**Methods:**

Data from a multi-centric, two-arm randomized controlled trial (RCT) was analyzed, including 210 patients (F32, single episode: *n* = 72, F33, recurrent major depression: *n* = 135, F31-II, bipolar type II: *n* = 3) and 125 healthy controls. Waist circumference, body mass index, body fat, blood pressure, cholesterol levels, triglycerides, and blood glucose were considered as cardiovascular risk markers. CRF was assessed with a submaximal ergometer test. Differences between groups were examined via *χ*^2^-tests and (multivariate) analyses of covariance.

**Results:**

Compared to healthy controls, patients with depression had a higher cardiovascular risk as evident from about half of the examined indicators. In the total sample, participants with good CRF had more favourable scores across nearly all risk markers than counterparts with poor CRF. For most variables, no interaction occurred between group and fitness, indicating that in patients and controls, similar differences existed between participants with poor and good CRF. Few differences in risk markers were found between patients with mild, moderate and severe depression, and no interaction occurred between depression severity and CRF.

**Discussion:**

Patients with depression and healthy controls differ in several cardiovascular risk markers, putting patients at increased risk for CVDs. In contrast, people with good CRF show more favourable cardiovascular risk scores, a relationship which was observed in both healthy controls and patients with depression. Physical health of psychiatric patients should receive the clinical attention that it deserves. Lifestyle interventions targeting healthy diet and/or physical activity are recommended as a physically active and healthy lifestyle contributes equally to patients’ mental well-being and cardiovascular health.

## 1. Introduction

Depression is a global health concern, affecting millions of people worldwide (1). Depending on the geographic region, estimates of lifetime prevalence of major depressive disorder vary between 2 and 30% (2–5). Depression is more prevalent among women than men (about 2:1 ratio), and the median age of onset is around 25 years (6, 7). Depression strongly contributes to higher Disability Adjusted Life Years (DALYs) (8). Projections assume that depression will be the leading cause of disability worldwide by 2030 (9), which might be partly due to the high comorbidity with cardiovascular diseases (CVDs) (10, 11).

Close links between the mind and the heart have been suggested already in the first half of the 17th century, but empirical evidence for this assumption only appeared around 1930 when researchers found that patients who were depressed at the time they experienced an acute myocardial infarction had a significantly higher mortality risk compared to non-depressed counterparts (6, 12). Since then, research on the associations between symptoms of depression, CVDs and mortality steadily increased (13).

With regard to excess mortality, compared to the general population, individuals with severe mental illnesses (SMI) die on average 10–25 years earlier, most often in close association with CVDs (10, 14, 15). This mortality gap appeared to increase over the past years (16). Even in Scandinavia, where high-quality and most equitable healthcare is provided, this gap has not substantially narrowed during the past decades (17). One reason for the persisting or widening gap might be that secondary prevention is much less successful in people with mental illnesses than in the general population including programs that target on reduction of overweight, smoking, unhealthy nutrition and physical inactivity (10).

In line with this, compared to the general population, individuals with depression have an increased risk for nearly all ‘traditional’ cardiovascular risk factors, including higher prevalence of overweight/obesity, type II diabetes, hypertension, dyslipidaemia, inflammation, autonomic dysfunction, unbalanced diet, higher alcohol consumption, tobacco smoking, and low compliance to pharmacological and non-pharmacological treatments as well as higher levels of sedentary behavior (7, 18). This notion was supported by a recent review (13), in which a large number of meta-analyses were identified on the relationship between depression and excess mortality (*k* = 14), CVDs (*k* = 15), diabetes (*k* = 25), overweight/obesity (*k* = 15), and metabolic syndrome (*k* = 5). In summary, the association between depression and excess mortality is considered as a robust epidemiological finding, which is partly attributable to the fact that people with depression are at increased risk for CVDs and accumulate more cardiovascular risk factors. However, the causal associations between depression and CVDs are complex and to some extent bidirectional. For instance, while depression is associated with incident CVD, depression can also be a consequence of cardiac events and worsen prognosis.

Among individuals with depression, biological and behavioral factors may simultaneously contribute to the increased risk of CVD (7, 10, 16, 17). Biological mechanisms include alterations in heart function and rhythm, increased coagulability and platelet aggregation, dysregulations of the autonomic nervous system, alterations of the hypothalamic–pituitary–adrenocortical (HPA) axis, increased inflammation, and endothelial dysfunction (6, 7, 12, 19–21). Further, psychopharmacological treatments can lead to weight gain and metabolic changes (6, 21), as they contribute to obesity, dyslipidaemia, type II diabetes and other metabolic alterations (22, 23). Another factor is seen in the decreased likelihood of receiving adequate medical care (19, 23). Psychiatrists and family physicians might be more likely to oversee and/or not treat physical conditions in individuals with depression, because they might wrongly assume that physical symptoms are psychological (diagnostic overshadowing) or because individuals with depression are less able or likely to communicate their physical needs (17, 24). Although screening recommendations have existed for almost 20 years, many patients with psychiatric disorders remain unscreened with regard to cardiovascular risk factors (17, 25). Moreover, given that socioeconomic disadvantage is more common among people with SMI (16), costs associated with insurance and treatment may be further barriers (24). Finally, poor compliance with healthy lifestyle recommendations such as balanced diet, avoiding tobacco, and sufficient and regular physical activity might also contribute to pathophysiology of CVDs in people with depression (6, 7, 19, 21).

So far, most public health efforts to reduce mortality in people with SMI have been directed towards reducing suicide rates, although a large number of excess deaths are the result of chronic physical health conditions (16, 26). Nevertheless, awareness for more systematic screening, monitoring and management of existing CVDs and their risk factors in this patient population is increasing (10, 25). This may help to identify individuals with particularly high risks who need different monitoring and/or intervention schemes. Moreover, systematic assessment of modifiable risk factors (see below) that moderate the CVD risk may help to identify areas in which there is a particularly great need for action (10).

Level of lifestyle physical activity and cardiorespiratory fitness (CRF) might be such moderating factors (7). Caspersen et al. (27) defined physical activity as “any bodily movement produced by skeletal muscles that results in energy expenditure” (p. 126), whereas CRF corresponds to one specific component of health-related physical fitness (an attribute that people have or achieve). According to Caspersen et al. (27), CRF relates to the “ability of the circulatory and respiratory systems to supply fuel during sustained physical activity and to eliminate fatigue products after supplying fuel” (p. 128). The reasons why physical activity and CRF might qualify as moderating factors are as follows: first, people with depression are less likely to engage in regular physical activity, and tend to engage in more sedentary activities (7). Second, CRF is significantly decreased in people with depression (28), and significantly reduced heart rate recovery further points towards autonomic dysfunction in these patients (29). Third, poor CRF is a risk factor for CVDs and all-cause mortality (30), and improvements are associated with a reduced risk, independent of other relevant factors such as age, body composition and smoking (31). Importantly, CRF improves in a clinically meaningful way in people with depression after participation in exercise training (32, 33). Fourth, empirical evidence suggests that the CVD risk of depression is mitigated in people who engage in regular physical activity or who present with better CRF. To provide some examples, the prospective Cooper Center Longitudinal Study with 47’702 adults showed that both history of depression (HR = 1.24) and metabolic syndrome (HR = 1.28) were independent risk factors of increased mortality risk, with the highest risk found in individuals combining both conditions (HR = 1.59). By contrast, moderate (HR = 0.64) or high CRF (HR = 0.50) were associated with a significantly decreased mortality risk. Importantly, those who suffered from both depression and metabolic syndrome and showed moderate or high CRF levels had a similar mortality risk as controls with low CRF (34). Similarly, a longitudinal study with 5’888 individuals from US communities showed that individuals presenting with either depressive symptoms or low physical activity were at greater risk for premature cardiovascular mortality. The probability was even higher in those who combined both risk factors. After adjustment for further confounders, physical activity was associated with a 26% reduction of cardiovascular mortality. This rate was similar in people without and with previously established coronary heart disease (35).

Given the background presented above, the purpose of the present study was five-fold. First, to find out whether common physiological cardiovascular risk factors (waist circumference, body mass index, body fat, blood pressure, cholesterol levels, triglycerides, and blood glucose) differ between patients with depression and healthy (non-depressed) controls. Second, to examine whether patients and controls differ in CRF, and whether higher CRF is associated with a lower cardiovascular risk. Third, to examine whether cardiovascular risk is moderated by participants’ CRF level. Fourth, to examine whether within the patient sample, cardiovascular risk factors would differ between patients with mild, moderate and severe depression, after controlling for sociodemographic factors, depression history and antidepressant intake. Fifth, to examine whether the relationship between depressive symptom severity and cardiovascular risk is moderated by patients’ CRF levels.

Based on current empirical evidence, we formulated the following hypotheses. First, cardiovascular risk is increased in patients with depression compared to healthy controls (Hypothesis 1) (13). Second, healthy controls present with higher CRF than patients with depression (Hypothesis 2a); participants with better CRF have lower cardiovascular risk (Hypothesis 2b) (28). Third, differences in cardiovascular risk factors between patients with depression and healthy controls are more pronounced in participants with low CRF levels (Hypothesis 3) (34, 35). Fourth, patients with higher depressive symptom severity report a less favorable cardiovascular risk profile than counterparts with lower symptom severity (Hypothesis 4) (13). Fifth, depressive symptom severity and CRF interact, in the sense that stronger (positive) associations appear between symptom severity and cardiovascular risk in patients with poor CRF compared to counterparts with better CRF (Hypothesis 5) (34, 35).

## 2. Materials and methods

### 2.1. Design

The present paper is based on baseline data from a multi-centric, two-arm randomized controlled trial (RCT), including an intervention group (personalized physical activity and exercise counselling program) and a placebo control group (general instructions about health-enhancing physical activity). The study was initiated by the Department of Sport, Exercise and Health of the University of Basel, and carried out in cooperation with four Swiss psychiatric clinics (2 public, 2 private).

### 2.2. Participants

Recruitment lasted from June 2019 to October 2021. Based on a structured clinical interview, all participants fulfilled ICD-10 diagnosis for first (F32) or recurrent depression (F33) and bipolar disorder type II, currently depressed (F31-II). Moreover, information was collected on duration of the current depressive episode, number of previous depressive episodes, as well as psychiatric and somatic comorbidities. As part of the screening process, patients completed the 21-item Beck Depression Inventory-II (BDI-II) (36, 37) to assess depressive symptom severity. Subjective information on physical activity during the last week before entering the clinic was collected via the short version of the International Physical Activity Questionnaire (IPAQ) (38). To be eligible for the present study, patients had to meet the following inclusion criteria: (a) 18–65 years of age, (b) presence of major depression according to ICD-10 diagnostic criteria (F32, F33) or bipolar disorder type II, currently depressed (F31-II), (c) BDI ≥ 17 (at least borderline clinical depression), (d) currently not meeting the American College of Sports Medicine (ACSM) physical activity recommendations (IPAQ < 150 min/week of moderate-to-vigorous physical activity), (e) written informed consent, and (f) ability to speak and read German. An age-and gender-matched sample of healthy controls was aimed at to allow an unbiased comparison of cardiovascular risk markers with patients. Recruitment of healthy controls was done through advertisements in online forums and word-of-mouth recommendations. For healthy controls, the following inclusion criteria were applied: (a) women and men, (b) 18–65 years of age, (c) HAMD17 ≤ 7, (d) BDI ≤ 13, (e) currently not meeting the ACSM physical activity recommendations, (f) written informed consent, and (g) ability to speak and read German.

Recruitment was delayed due to COVID-19-related challenges and had to be finalized after 244 patients and 151 healthy controls had been recruited, with 210 patients and 125 healthy controls fulfilling all inclusion criteria and having valid data for depressive symptoms severity and CRF at baseline. Eight patients and two controls were excluded because of reported intake of betablockers, which may have had an effect on heart rate during the fitness test and blood pressure.

The study was reviewed by a competent ethics committee (Ethikkommission Nordwest-und Zentralschweiz; ref. approval no. 2018-00976) and all procedures were in line with the ethical principles of the Declaration of Helsinki. The intervention study was registered in the WHO trial register (trial number: ISRCTN10469580). Participants received information about the general goals of the study and provided informed written consent before study entry. Participation in the study was voluntary and withdrawal or discontinuation possible at any time.

### 2.3. Data assessment and measures

Screening of patients took place in the first week after admission to in-patient treatment, baseline data assessment after 2–3 weeks after admission in one of the four involved clinics. Screening and data assessment were done simultaneously in healthy controls. All data assessment procedures were identical for patients and controls.

The BDI-II (36, 37) was applied to assess depression severity. The 21-item BDI-II is a frequently used tool to assess symptoms of unipolar depression such as affective, behavioral, cognitive, and somatic symptoms (e.g., “I am so unhappy/sad that I cannot stand it”). Items were answered on a 4-point scale (from 0 to 3), resulting in sum scores from 0 to 63, with higher scores reflecting stronger depressive symptomatology. The reliability and validity of the BDI-II is well documented (39). Depression severity was defined as follows: mild depression (BDI-II = 0–19), moderate depression (BDI-II = 20–28) and severe depression (BDI-II = 29–63) (40).

CRF (VO_2_max) was estimated with the Åstrand indirect test of maximal oxygen uptake (41). The test was performed on a bicycle ergometer (Bike Forma; Technogym, Lyss, Switzerland) at the same time of the day (starting between 8–10 am). The pedalling frequency was set at 50 revolutions per minute (rpm), while the workload was adjusted so that the heart rate was kept between 130–160 beats per minute (bpm) in participants younger than 40 years old and between 120–150 bpm in participants older than 40 years old. The Borg Rating of Perceived Exertion scale (42) was used to ensure that participants maintain their exercise intensity level at 13 or 14 (slightly strenuous). Following stabilization of heart rate after 5 or 6 min, peak oxygen uptake (l min) was estimated based on mean steady-state, sex and power-output, using a nomogram (41) and including a correction factor for age. Oxygen uptake was expressed as VO_2_max (ml/kg/min), after correction for body weight. Gender and age-adjusted cut-offs were used based on norms defined by the ACSM to categorize participants into groups with poor-to-fair (labelled as “poor”) and good-to-superior CRF (labelled as “good”) (43). Previous studies have demonstrated the reliability and validity of the Åstrand nomogram and the linear extrapolation for deriving VO_2_max (44).

A digital weighing scale (BC-545; Tanita, Arlington Heights, Illinois, United States) was used to measure body weight (to the nearest 0.1 kg, in light cloths and without shoes). Body height was measured with a stadiometer (to the nearest 0.5 cm, without shoes). Body Mass Index (BMI) was calculated as: weight (kg)/(standing height [meters (m)]^2^). Participants were classified as overweight if their BMI was ≥25.0 kg/m^2^, and obese if their BMI was ≥30.0 kg/m^2^ (45). Percentage of body fat was also measured with the BC-545 weighing scale via bioelectrical impedance analysis. Following WHO standards (45), maximum levels of 32% for women and 25% for men are recommended. A flexible tape at the natural waist (half way between the ribcage and the iliac crest) was used to determine waist circumference. The expert panel of the National Cholesterol Education Program III (46) defines a waist circumference of ≥80 cm (women) and ≥94 cm (men) as a risk factor for metabolic syndrome.

After a 5 min resting period, systolic (SBP) and diastolic (DBP) blood pressure were measured on the left upper arm, in a seated position. Blood pressure was assessed twice within 5 min with the Omron^®^ digital blood pressure monitor. Previous studies have supported the validity of this oscillometric device (47). Participants were considered hypertensive if they had SBP of ≥140 mmHg and/or DBP of ≥90 mmHg (48).

Capillary blood was drawn between 07:00 and 08:30 after fasting since 22:00 the day before by trained research assistants. Total cholesterol [TC], low-density-lipoprotein cholesterol [LDL-C], high-density-lipoprotein cholesterol [HDL-C], triglycerides [TG] and HbA1c were analyzed via the Afinion test (Alere Technologies; Abbott, Wädenswil, Switzerland). One drop of blood was taken up by the test strip and read by the machine. Alere point-of-care (PAC) analyser results showed good correspondence with reference laboratory tests for HbA1c and lipid levels (49, 50). The following cut-offs of the National Cholesterol Education Program III were considered for borderline high total cholesterol (≥5.14 mmoL/L), low HDL (≤1.54 mmoL/L), borderline high LDL cholesterol (≥3.34 mmoL/L) and borderline high triglycerides (≥1.69 mmoL/L) (46). HbA1c scores of ≥5.70 were considered as cut-point for prediabetes (51, 52).

Participants were further asked to report their sex, age, language, nationality, marital status, level of education, employment (rate) (in patients prior to hospitalization), years of job experience, and the number of children living at home. Information about smoking status was collected as part of the clinical interview with a simple yes/no question (Are you currently smoking? Yes = 1, no = 0). Additionally, in patients, information about duration of current depressive episode, number of prior depressive episodes, age of onset of depression, and current medication was assessed via clinical interview.

### 2.4. Statistical analyses

Sample characteristics are presented as M (mean), SD (standard deviation), % (percentage), and *n* (frequencies). Descriptive statistics (*M*, *SD*, %, *n*) for the cardiovascular risk markers are reported separately for the total sample, patients vs. healthy controls, poor-fair and good-superior CRF, and patients with low, moderate or severe depression severity. Differences between these groups were tested via analyses of variance for metric outcomes. Differences between dichotomized outcomes (risk factor not present = 0, present = 1) were tested via *χ*^2^-tests. To examine interactions and to control for possible confounders, a multivariate (2-way) analysis of covariance with group (patients vs. controls) and CRF (poor vs. good) as fixed factor and the interaction term (group^*^CRF) was calculated. Covariates were considered only if they were significantly associated with the outcomes in the (M)ANCOVAS. The MANCOVAs were followed by univariate analyses. (M)ANCOVAs were also performed within the patient-sample, to examine differences based on symptom severity. As three groups were compared, Bonferroni post-hoc tests were applied. Again, covariates were considered only if they were significantly associated with the outcomes in the (M)ANCOVAS. Moreover, these analyses were also controlled for disease history (recurrent vs. first-episode), and medication (antidepressants vs. no antidepressants). All analyses were calculated with SPSS 28 (IBM Corporation, Armonk, NY, United States), and the level of statistical significance was set at *p* < 0.05 across all analyses.

## 3. Results

### 3.1. Sample characteristics of patients and controls

Table 1 provides an overview of the sample characteristics of the total sample, patients and controls. Sex was equally distributed (53.7% female). The mean age was 38.82 years. Most of the participants spoke German as first language (86.6%), reported Swiss nationality (74.3%), and were single (69.9%). About half of the sample reported higher education (48.1%), and one third (33.4%) was smoking. In the patient sample, approximately one third (34.4) had a F32 diagnosis (first-episode), whereas two thirds (64.3%) had recurrent major depression (F33 diagnosis). Only *n* = 3 had a bipolar-II, currently depressed diagnosis. At baseline (2–3 after admission to the hospital), 41.0% of the patients reported low symptom severity, whereas 34% reported moderate and 24.3% severe depression. Moreover, 88.1% of the patients were treated with antidepressants. CRF was relatively low in the overall sample, with 54.3% of the participants achieving only poor CRF. Although we attempted to recruit a sex-and age-matched control sample, healthy controls turned out to be younger than patients. Table 1 further shows that patients reported Swiss nationality more often, were less likely to report higher education, were more likely to smoke, and had higher body weight. Since patients were older than controls, they also reported more years of job experience. Finally, compared to controls, patients achieved lower VO_2_max scores.

**Table 1:** Sample characteristics, and group differences, based on *χ*^2^-tests and ANOVA.

	Total sample (*N* = 335)		Patients with depression (*n* = 210)		Healthy controls (*n* = 125)		*χ*^2^-tests and ANOVA	
	*n*	%	*n*	%	*n*	%	*χ* ^2^	*ϕ*
Sex (female)	180	53.7	111	52.9	69	55.2	0.18	0.023
Language (German as first language)	280	86.6	183	87.1	107	85.6	0.16	0.022
Nationality (Swiss)	249	74.3	172	81.9	77	61.6	16.93^***^	0.225
Marital status (single)	234	69.9	154	73.3	80	64.0	3.24	0.072
Level of education (higher education)	161	48.1	75	35.7	86	68.8	34.36^***^	0.320
Children living at home (yes)	77	23.0	50	23.8	27	21.6	0.22	0.025
Antidepressant intake (yes)	185	55.2	185	88.1	0	0.0	245.93^***^	0.857
Smoking (yes)	112	33.4	86	41.0	26	20.8	14.30^***^	0.207
Depression subtype								
Single episode (F32)	–	–	72	34.3	–	–	–	–
Recurrent major depression (F33)	–	–	135	64.3	–	–	–	–
Bipolar disorder type II (F31-II)	–	–	3	1.4	–	–	–	–
Cardiorespiratory fitness								
Poor	182	54.3	126	60.0	56	44.8	7.30^**^	0.148
Good	153	45.7	84	40.0	69	55.2		
	*M*	*SD*	*M*	*SD*	*M*	*SD*	*F*	*η* ^2^
Age (in years)	38.82	13.19	40.60	12.06	35.82	13.64	10.60^**^	0.031
Height (in cm)	171.49	9.31	171.63	9.51	171.28	9.00	0.11	0.000
Weight (in kg)	76.57	19.92	80.23	20.89	70.44	16.51	20.02^***^	0.057
Employment rate (in %)	24.31	20.14	23.86	21.14	25.08	18.39	0.29	0.001
Years of job experience (in years)	16.24	13.03	17.95	12.50	13.37	13.46	9.91^**^	0.029
Estimated VO_2_max (mL/kg/min)	35.02	9.88	33.13	9.48	38.18	9.78	21.71^***^	0.061
Depressive symptom severity (at baseline)	15.41	12.13	22.13	10.31	4.11	3.42	358.16^***^	0.518
Duration of current episode (in weeks)^a^	–	–	38.92	51.83	–	–	–	–
Number of prior depressive episodes^b^	–	–	3.11	5.97	–	–	–	–
Age of onset of depression (in years)^c^	–	–	29.52	14.13	–	–	–	–

VO_2_max, maximal oxygen uptake. *p < 0.05, **p < 0.01, and ***p < 0.001.

a 29 values missing.

b 15 values missing.

c 14 values missing.

### 3.2. Cardiovascular risk markers in patients versus controls

Table 2 shows that patients had higher BMI, waist circumference and body fat than controls. Patients had also higher LDL cholesterol values and higher HbA1c scores. Using dichotomized variables, patients were more likely to be overweight/obese, to have high waist circumference, high body fat, high total cholesterol and LDL cholesterol levels, whereas they were more likely to have low HDL cholesterol levels.

**Table 2:** Descriptive statistics and group differences based on ANOVA and *χ*^2^-tests between patients with depression and healthy controls (uncontrolled).

		Total sample (*N* = 335)		Patients with depression (*n* = 210)		Healthy controls (*n* = 125)		ANOVA and *χ*^2^-tests	
	*N*	*M*	*SD*	*M*	*SD*	*M*	*SD*	*F*	*η* ^2^
Body Mass Index (BMI) (in m/kg^2^)	333	25.87	5.82	27.01	6.08	23.94	4.77	23.08^***^	0.065
Waist circumference (in cm)	335	87.27	17.85	90.17	17.86	82.41	16.80	15.48^***^	0.044
Body fat (in %)	332	28.47	10.09	29.88	10.42	26.13	9.08	11.05^***^	0.032
Systolic blood pressure (in mmHG)	335	115.93	14.17	116.59	13.81	114.82	14.76	1.22	0.004
Diastolic blood pressure (in mmHG)	335	76.95	9.51	76.81	9.79	77.18	9.05	0.12	0.000
Total cholesterol (in mmol/L)	312	4.90	1.20	4.97	1.15	4.78	1.27	1.67	0.005
HDL cholesterol (in mmol/L)	306	1.43	0.43	1.40	0.43	1.49	0.41	3.01	0.010
LDL cholesterol (in mmol/L)	302	2.82	0.96	2.94	0.91	2.62	1.00	7.86^**^	0.026
Triglycerides (in mmol/L)	312	1.50	1.22	1.43	1.08	1.63	1.44	1.93	0.006
HbA1c (in %)	320	5.32	0.48	5.39	0.55	5.21	0.32	11.07^***^	0.034
	*N*	*n*	%	*n*	%	*n*	%	*χ* ^2^	*ϕ*
Overweight/obesity	333	164	49.2	125	59.8	39	31.5	25.04^***^	0.274
High waist circumference	335	146	43.6	109	51.9	37	29.6	15.86^***^	0.218
High body fat	332	155	46.7	114	54.8	41	26.5	14.76^***^	0.211
Hypertension	335	44	13.1	28	13.3	16	12.8	0.02	0.008
Borderline high total cholesterol	312	112	35.9	80	40.2	32	28.3	4.42^*^	0.119
Low HDL cholesterol	306	49	16.0	38	19.3	11	10.1	4.41^*^	0.120
Borderline high LDL cholesterol	302	80	26.5	60	30.9	20	18.5	5.49^*^	0.135
Borderline high triglycerides	312	84	26.9	49	24.6	35	31.0	1.48	0.069
High HbA1c scores (prediabetic/diabetic)	320	37	11.6	28	14.0	9	7.5	3.10	0.098

HDL, high density lipoprotein; LDL, low density lipoprotein; HbA1c, glycated hemoglobin. Differences in *N* due to missing values in some of the outcome measures. *p < 0.05, **p < 0.01, and ***p < 0.001.

### 3.3. Cardiovascular risk markers in participants with poor and good fitness

As shown in Table 3, participants with good fitness had more favorable scores on all cardiovascular risk makers. Thus, they had lower BMI, waist circumference, body fat, systolic and diastolic blood pressure, total cholesterol, LDL cholesterol, triglycerides and HbA1c, whereas they had higher HDL cholesterol concentrations. These differences were corroborated in the analyses based on the dichotomized variables.

**Table 3:** Descriptive statistics and group differences based on ANOVA and *χ*^2^-tests between individuals with poor and good CRF (uncontrolled).

		Poor CRF (*n* = 153)		Good CRF (*n* = 182)		ANOVA and *χ*^2^-tests	
	*N*	*M*	*SD*	*M*	*SD*	*F*	*η* ^2^
Body Mass Index (BMI) (in m/kg^2^)	333	28.00	6.23	23.33	4.03	63.26^***^	0.160
Waist circumference (in cm)	335	93.19	17.33	80.24	15.83	50.21^***^	0.131
Body fat (in %)	332	30.96	10.20	25.54	9.16	25.54^***^	0.072
Systolic blood pressure (in mmHG)	335	119.96	14.37	111.15	12.39	35.41^***^	0.096
Diastolic blood pressure (in mmHG)	335	79.90	9.73	73.44	7.94	43.28^***^	0.115
Total cholesterol (in mmol/L)	312	5.04	1.27	4.75	1.09	4.59^*^	0.015
HDL cholesterol (in mmol/L)	306	1.34	0.40	1.54	0.43	17.70^***^	0.055
LDL cholesterol (in mmol/L)	302	2.99	1.01	2.64	0.86	10.29^***^	0.033
Triglycerides (in mmol/L)	312	1.66	1.27	1.33	1.14	5.77^*^	0.018
HbA1c (in %)	320	5.40	0.59	5.23	0.29	10.54^***^	0.032
	*N*	*n*	%	*n*	%	*χ* ^2^	*ϕ*
Overweight/obesity	333	122	67.4	42	27.6	52.29^***^	0.396
High waist circumference	335	112	61.5	34	22.2	52.27^***^	0.395
High body fat	332	113	62.8	42	27.6	40.90^***^	0.351
Hypertension	335	40	22.0	4	2.6	27.32^***^	0.286
Borderline high total cholesterol	312	68	41.5	44	29.7	4.66^*^	0.122
Low HDL cholesterol	306	37	23.0	12	8.3	12.27^***^	0.200
Borderline high LDL cholesterol	302	52	33.1	28	19.3	7.38^**^	0.156
Borderline high triglycerides	312	58	35.4	26	17.6	12.53^***^	0.200
High HbA1c scores (prediabetic/diabetic)	320	27	15.9	10	6.7	6.62^**^	0.144

CRF, cardiorespiratory fitness; HDL, high density lipoprotein; LDL, low density lipoprotein; HbA1c, glycated hemoglobin. Differences in *N* due to missing values in some of the outcome measures. *p < 0.05, **p < 0.01, and ***p < 0.001.

### 3.4. Differences between patients and controls, dependent on their fitness level

**Figure 1:**
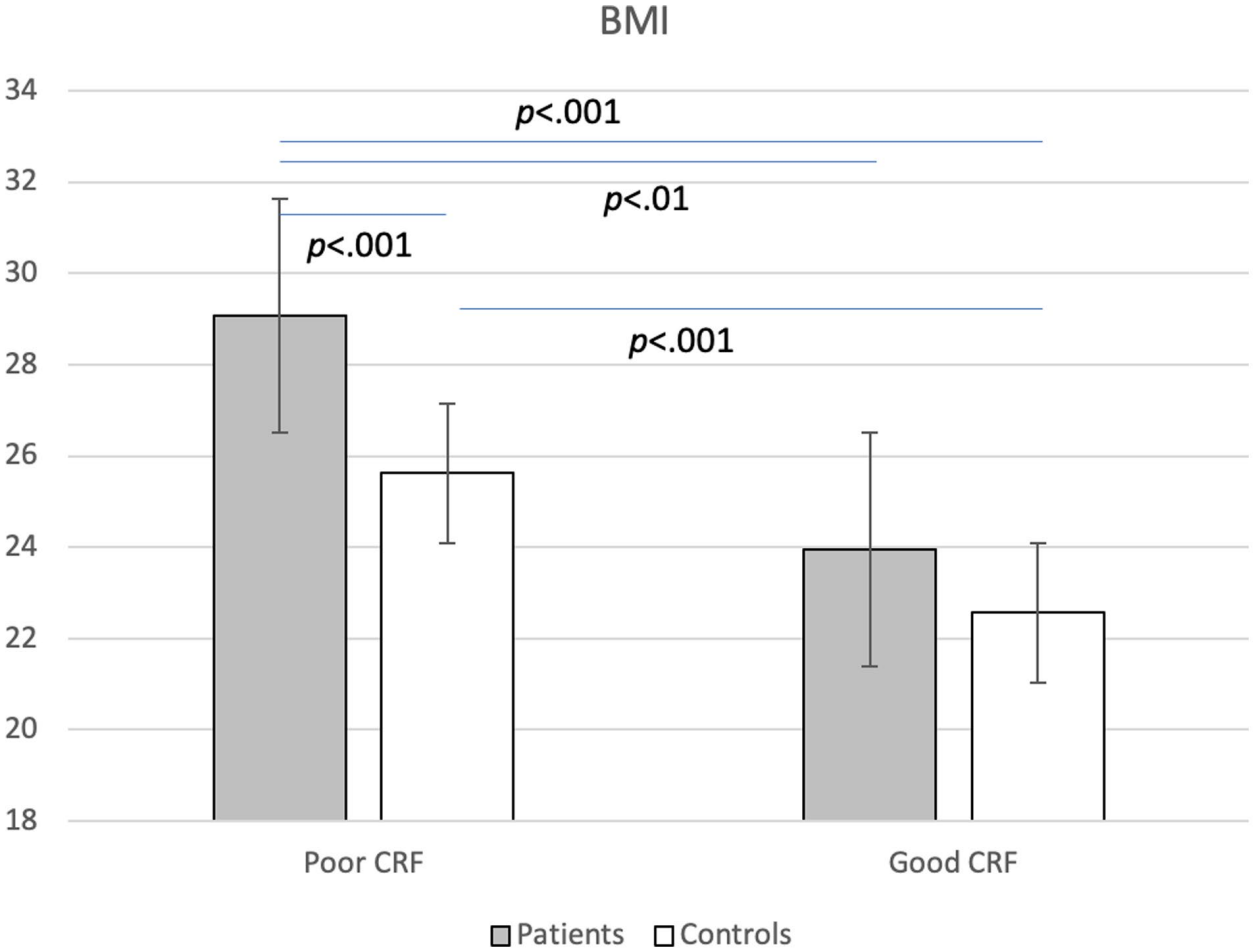
Interaction between group (patients vs. controls) and cardiorespiratory fitness (CRF: poor vs. good) on body mass index (BMI).

A MANCOVA considering all cardiovascular risk markers and all statistically significant covariates (age, smoking) yielded a significant main effect for group, Wilks-Lamda: *F*(10,279) = 3.67, *p* < 0.001, *η*^2^ = 0.116, and fitness, Wilks-Lamda: *F*(10,279) = 6.71, *p* < 0.001, *η*^2^ = 0.194, whereas no significant interaction effect was observed, Wilks-Lamda: *F*(10,279) = 1.26, *p* = 0.255, *η*^2^ = 0.043. Table 4 shows the findings of the univariate ANCOVAS, which generally corroborate the results of the uncontrolled ANOVAs reported in Tables 2, 3. Thus, after controlling for relevant covariates, and after simultaneously considering the effects of group and CRF, we found more favorable cardiovascular risk profiles in controls and in participants with good CRF. The only significant interaction was observed for BMI, showing that among participants with poor CRF, patients had significantly higher BMI scores than controls, which was not the case in participants with good CRF (Figure 1). Thus, among participants with poor CRF, the group mean BMI of patients was close to the cut-point for obesity (*M* = 28.96, *SD* = 4.50), whereas in healthy controls, the group mean score was only slightly above the cut point for overweight (*M* = 25.72, *SD* = 5.32). Table 4 also shows the distribution of risk factors (after dichotomization) for the four different groups. As a general pattern, presence of risk factors was most frequently observed in the group of patients with poor CRF (with the exception of hypertension and triglycerides).

**Table 4:** Main and interaction effects of two-way ANOVA and *χ*^2^-tests, with group (patients vs. controls) and CRF (poor vs. good) as fixed factors.

	Group		CRF		Group^*^CRF	
	*F*	*η* ^2^	*F*	*η* ^2^	*F*	*η* ^2^
Body Mass Index (BMI)	14.59^***^	0.043	40.82^***^	0.111	6.23^***^	0.019
Waist circumference	7.69^**^	0.023	33.77^***^	0.093	1.34	0.004
Body fat	7.04^**^	0.021	13.53^***^	0.040	3.60	0.011
Systolic blood pressure	2.27	0.007	22.87^***^	0.065	0.31	0.001
Diastolic blood pressure	6.27^*^	0.019	31.89^***^	0.089	3.76	0.011
Total cholesterol	0.01	0.000	1.86	0.006	0.02	0.000
HDL cholesterol	0.48	0.002	14.40^***^	0.046	0.11	0.000
LDL cholesterol	2.34	0.008	6.05^*^	0.020	0.10	0.000
Triglycerides	2.91	0.009	6.00^*^	0.019	3.82	0.012
HbA1c	4.69^*^	0.015	3.87^*^	0.012	1.25	0.004

	Patients, poor CRF *n* (%)^a^	Patients, good CRF *n* (%)^a^	Controls, poor CRF *n* (%)^a^	Controls, good CRF *n* (%)^a^	*χ*^2^ (*ϕ*)
Overweight/obesity	96 (76.8)	29 (34.5)	26 (46.4)	13 (19.1)	70.13 (0.459)^***^
High waist circumference	87 (69.0)	22 (26.2)	25 (44.6)	12 (17.4)	62.84 (0.433)^***^
High body fat	86 (69.4)	28 (33.3)	27 (48.2)	14 (20.6)	50.28 (0.389)^***^
Hypertension	27 (21.4)	1 (1.2)	13 (23.2)	3 (4.3)	27.76 (0.288)^***^
Borderline high total cholesterol	51 (44.0)	29 (34.9)	17 (35.4)	15 (13.4)	7.96 (0.160)^*^
Low HDL cholesterol	30 (26.3)	8 (9.6)	7 (14.9)	4 (6.5)	15.77 (0.227)^**^
Borderline high LDL cholesterol	40 (36.0)	20 (24.1)	12 (26.1)	8 (12.9)	11.32 (0.194)^**^
Borderline high triglycerides	39 (33.6)	10 (12.0)	19 (39.6)	16 (24.6)	16.07 (0.227)^***^
High HbA1c scores (prediabetic/diabetic)	20 (17.2)	8 (9.5)	7 (13.0)	2 (3.0)	8.80 (0.166)^*^

CRF, cardiorespiratory fitness; HDL, high density lipoprotein; LDL, low density lipoprotein; HbA1c, glycated hemoglobin. In the ANCOVAs, covariates were only considered if they were significantly associated with the outcome. BMI: nationality, age. Waist circumference: nationality, age. Body fat: age, smoking. Systolic blood pressure: education, age. Diastolic blood pressure: age. Total cholesterol: age. HDL cholesterol: smoking. LDL cholesterol: age. Triglycerides: age. Glucose: age. *p < 0.05, **p < 0.01, and ***p < 0.001.

a Percentages describe portion of participants in this group with cardiometabolic risk factor present.

### 3.5. Cardiovascular risk markers in patients with mild, moderate, and severe depression

Table 5 shows that only one cardiovascular risk marker (HbA1c) differed between patients with mild, moderate and severe depression. For HbA1c, scores were significantly higher in patients with severe symptoms compared to counterparts with mild or moderate depression. After dichotomization, no significant group differences were observed in any of the variables.

**Table 5:** Descriptive statistics and group differences based on ANOVA and *χ*^2^-tests between patients with low, moderate or severe depression (uncontrolled).

		Depresssive symptom severity							
		Mild (*n* = 87)		Moderate (*n* = 72)		Severe *(n* = 51)		ANOVA and *χ*^2^-tests	
	*N*	*M*	*SD*	*M*	*SD*	*M*	*SD*	*F*	*η* ^2^
Body Mass Index (BMI) (in m/kg^2^)	209	26.81	5.71	26.72	6.27	27.78	6.48	0.53	0.005
Waist circumference (in cm)	210	89.22	13.45	90.30	20.09	91.62	21.11	0.29	0.003
Body fat (in %)	208	28.74	10.67	29.72	10.67	32.05	9.42	1.63	0.016
Systolic blood pressure (in mmHG)	210	117.53	14.59	116.54	12.18	115.07	14.71	0.51	0.005
Diastolic blood pressure (in mmHG)	210	76.91	9.57	76.86	9.87	76.57	10.24	0.02	0.000
Total cholesterol (in mmol/L)	199	4.90	1.13	4.98	1.17	5.04	1.71	0.22	0.002
HDL cholesterol (in mmol/L)	197	1.37	0.42	1.45	0.44	1.37	0.44	0.81	0.008
LDL cholesterol (in mmol/L)	194	2.89	0.90	2.98	0.94	2.96	0.92	0.19	0.002
Triglycerides (in mmol/L)	199	1.48	1.06	1.29	1.00	1.54	1.20	0.90	0.009
HbA1c (in %)	200	5.36^a^	0.34	5.27^b^	0.28	5.60^a,b^	0.94	5.48^**^	0.053
	*N*	*n*	%	*n*	%	*n*	%	*χ* ^2^	*ϕ*
Overweight/obesity	209	53	60.9	39	54.2	33	67.3	2.10	0.101
High waist circumference	210	42	48.3	36	50.0	31	60.8	2.17	0.102
High body fat	208	41	47.7	39	54.2	34	68.0	5.29	0.160
Hypertension	210	13	16.1	8	11.1	6	11.8	0.99	0.069
Borderline high total cholesterol	199	30	37.5	29	42.0	21	42.0	0.41	0.045
Low HDL cholesterol	197	17	21.3	9	13.2	12	24.5	2.65	0.116
Borderline high LDL cholesterol	194	24	30.8	20	29.4	16	33.3	0.20	0.032
Borderline high triglycerides	199	16	20.0	17	24.6	16	32.0	2.39	0.110
High HbA1c scores (prediabetic/diabetic)	200	10	12.3	8	11.4	10	20.4	2.24	0.106

HDL, high density lipoprotein; LDL, low density lipoprotein; HbA1c, glycated hemoglobin. Differences in *N* due to missing values in some of the outcome measures. ^a,b^Groups with equal superscript letters significantly differ from each other in Bonferroni post-hoc tests. *p < 0.05, **p < 0.01, and ***p < 0.001.

### 3.6. Differences between patients with different symptom severity, dependent on their fitness level

**Figure 2:**
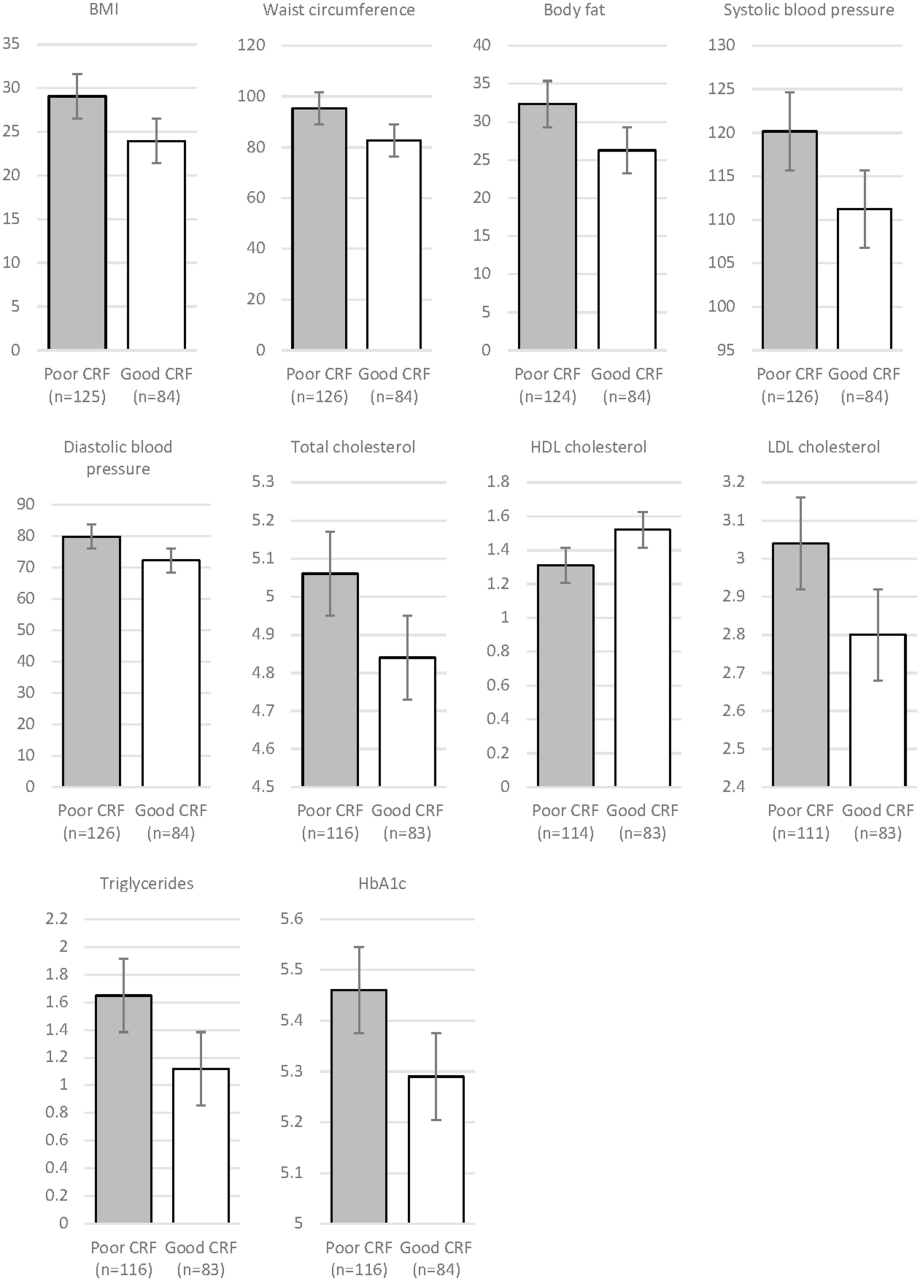
Differences in cardiovascular risk markers between patients with poor vs. good CRF.

A MANCOVA considering all cardiovascular risk markers and all statistically significant covariates (age, sex) yielded a significant main effect for fitness, Wilks-Lamda: *F*(10,173) = 6.52, *p* < 0.001, *η*^2^ = 0.274, but not for group, Wilks-Lamda: *F*(20,346) = 1.38, *p* = 0.129, *η*^2^ = 0.074. Moreover, no significant interaction effect occurred, Wilks-Lamda: *F*(20,346) = 0.77, *p* = 0.745, *η*^2^ = 0.043. Table 6 shows the findings of the univariate tests. Thus, after controlling for relevant covariates, and after simultaneously considering the effects of depressive symptom severity and CRF, we found more favorable cardiovascular risk profiles in participants with good CRF, whereas difference between patients with mild, moderate and severe depression were largely absent. Differences between patients with poor vs. good CRF are illustrated in Figure 2. As in the total sample, patients with good CRF had more favorable cardiovascular risk profiles than patients with poor CRF. A significant interaction was only found for systolic blood pressure. Unexpectedly, differences between participants with poor and good CRF were largest in patients with mild depression. Table 6 also shows the distribution of risk factors (after dichotomization) for the six different groups.

**Table 6:** Main and interaction effects of two-way ANOVA and *χ*^2^-tests, with group (low, moderate vs. severe depression) and CRF (poor vs. good) as fixed factors.

Metric variables	Group		CRF		Group^*^CRF	
	*F*	*η* ^2^	*F*	*η* ^2^	*F*	*η* ^2^
Body Mass Index (BMI)	0.67	0.007	37.17^***^	0.157	0.03	0.000
Waist circumference	1.52	0.15	23.32^***^	0.105	0.52	0.005
Body fa	1.57	0.016	41.79^***^	0.174	0.01	0.000
Systolic blood pressure	0.21	0.002	11.62^***^	0.055	3.14^*^	0.030
Diastolic blood pressure	0.84	0.008	32.76^***^	0.140	0.39	0.004
Total cholesterol	1.04	0.011	0.96	0.005	0.47	0.005
HDL cholesterol	0.23	0.002	8.05^**^	0.041	0.03	0.000
LDL cholesterol	0.92	0.010	2.37	0.012	0.43	0.005
Triglycerices	0.31	0.003	11.05^**^	0.054	0.57	0.006
HbA1c	3.36^*^	0.034	4.28^*^	0.022	2.21	0.023

Dichotomized variables	Mild symptoms, poor CRF *n* (%)	Mild symptoms, good CRF *n* (%)	Moderate symptoms, poor CRF *n* (%)	Moderate symptoms, good CRF *n* (%)	Severe symptoms, poor CRF *n* (%)	Severe symptoms, good CRF *n* (%)	*χ*^2^ (*ϕ*)
Overweight/obesity	41 (78.8)	12 (34.3)	27 (73.0)	12 (34.3)	28 (77.8)	5 (35.7)	37.69 (0.425)^***^
High waist circumference	35 (67.3)	7 (20.0)	26 (70.3)	10 (28.6)	26 (70.3)	5 (35.7)	38.32 (0.427)^***^
High body fat	32 (62.7)	9 (25.7)	27 (73.0)	12 (34.3)	27 (75.0)	7 (50.0)	30.20 (0.381)^***^
Hypertension	14 (26.9)	0 (0.0)	7 (18.9)	1 (2.9)	6 (16.2)	0 (0.0)	20.44 (0.312)^***^
Borderline high total cholesterol	19 (41.3)	11 (32.4)	15 (44.1)	14 (40.0)	17 (47.2)	4 (28.6)	2.64 (0.115)
Low HDL cholesterol	14 (30.4)	3 (8.8)	4 (12.1)	5 (14.3)	12 (34.3)	0 (0.0)	16.12 (0.286)^***^
Borderline high LDL cholesterol	16 (36.4)	8 (23.5)	12 (36.4)	8 (22.9)	12 (35.3)	4 (28.6)	3.34 (0.131)
Borderline high triglycerides	15 (32.6)	1 (2.9)	9 (26.5)	8 (22.9)	15 (41.7)	1 (7.1)	18.25 (0.303)^***^
High HbA1c scores (prediabetic/diabetic)	8 (17.4)	2 (5.7)	3 (8.6)	5 (14.3)	9 (25.7)	1 (7.1)	7.83 (0.198)

CRF, cardiorespiratory fitness; HDL, high density lipoprotein; LDL, low density lipoprotein; HbA1c, glycated hemoglobin. In the ANCOVAs, covariates were only considered if they were significantly associated with the outcome. BMI: age, civil status, children at home. Waist circumference: age, sex, language, nationality, employment rate. Body fat: age, sex, civil status, children at home. Systolic blood pressure: age, sex, education, employment rate. Diastolic blood pressure: age, sex, children at home. Total cholesterol: age. HDL cholesterol: sex, language, nationality, civil status, children at home. LDL cholesterol: age. Triglycerides: –. Glucose: age, nationality. *p < 0.05, **p < 0.01, and ***p < 0.001.

## 4. Discussion

The key findings of the present study are that compared to healthy controls, patients with depression had a higher cardiovascular risk as evident from about half of the examined indicators. Moreover, participants with good CRF had more favourable scores across nearly all risk markers than counterparts with poor CRF. For most variables, no interaction occurred between group and fitness, indicating that in patients and controls, similar differences existed between participants with poor and good CRF. Finally, within the patient sample, no major differences in the presented cardiovascular risk markers were found between patients with mild, moderate and severe depression.

Five hypotheses were formulated and each of them will now be addressed in turn. Our first hypothesis was that patients with depression have higher cardiovascular risk compared to healthy controls. This hypothesis was supported for five markers (waist circumference, body mass index, body fat, diastolic blood pressure, HbA1c), whereas no differences were found for systolic blood pressure, total, HDL and LDL cholesterol, as well as triglycerides. The present findings are in line with previous studies showing that patients with depression have a markedly higher burden of physical comorbidities compared to other age-gender-matched hospital patients (53). The increased presence of cardiovascular risk markers may explain why cardiovascular and all-cause mortality are higher in patients with depression than in the general population (6, 12). While the higher all-cause mortality in depressed people is to some extent due to higher death rates from unnatural causes such as suicides, accidents, homicides and alcohol misuse, the mortality rate is also increased for natural causes (7, 14, 24, 54). Some scientists have criticized that the persisting mortality gap denotes a cynical disregard for the lost lives among people with mental illnesses (17). Others have highlighted that the high prevalence of depression worldwide is alarming and should make people with depression a priority target population for public health strategies to prevent CVDs and mortality (7). In our study, the largest differences between patients and controls were found for BMI, waist circumference and body fat, which is in line with recent epidemiological studies in depression and bipolar disorder, especially among female participants (55, 56). This is important because depression and obesity are widely prevalent issues with considerable implications for public health (57). However, given the cross-sectional nature of our data, we need to be careful with inferring causal relationships. Thus, while it is possible that people with depression might be more likely to develop overweight through dysregulated stress systems, through unhealthy lifestyles or medication, it is also conceivable that overweight favours the development of depression through negative effects on self-image or somatic consequences (57).

Our second hypothesis was that patients would present lower CRF than healthy controls (Hypothesis 2a), and that better CRF would be associated with a more favorable cardiovascular risk factor profile (Hypothesis 2b). This hypothesis was supported by our data. Thus, our results are in line with both cross-sectional and longitudinal studies showing that people with higher CRF have lower risk of reporting/developing mental illnesses (28, 43, 58, 59). For instance, Kandola et al. (58) showed in a meta-analysis of longitudinal cohort studies that participants with low or medium CRF have a 47 and 23% increased likelihood of incidence of mental health disorders. Moreover, our findings corroborate previous studies showing that higher CRF levels are associated with decreased overall cardiovascular risk (60, 61), as well as presence of risk markers such as overweight (62, 63), hypertension (64, 65), dyslipidemia (66, 67), and diabetes (68, 69).

Only limited support was found for our third hypothesis, stating that differences in cardiovascular risk markers between patients and healthy controls would be more pronounced in participants with low CRF levels. This finding is at odds with previous studies showing that simultaneously suffering from mental disorders and having low CRF would exacerbate the risk for CVDs and CVD-related mortality (34, 35). Rather, our findings show that having poor CRF is similarly associated with higher presence of cardiovascular risk markers in both healthy people and patients with depression. This is encouraging as CRF turned out to be modifiable via regular exercise training in both healthy populations (70, 71) and people with mental illnesses (72, 73).

Our fourth hypothesis, that patients with more severe depression would report a less favorable cardiovascular risk profile than counterparts with mild or moderate depression, was not supported. Again, this is at odds with prior investigations in which patients with more severe depression were more likely to develop a CVD (9). Given this result, the prerequisites for our fifth hypothesis, that the association between depressive symptom severity and cardiovascular risk would be moderated by patients’ CRF level, were not given. We assume that this unexpected result is attributable to the fact that our sample was relatively homogeneous (all in-patients), whereas previous cohort studies were based on broader populations. However, this finding could also be due to the fact that symptom severity assessed in this study reflects a momentary state. Maybe different results would have appeared if the longitudinal course of the disease had been considered.

While the focus of this paper was on biological risk markers, it should be noted that in our study the prevalence of smoking was higher in patients with depression (41.0%) than in healthy controls (20.8%). Accordingly, smoking was considered as a potential covariate in the subsequent (M)ANCOVAs. This finding supports prior research showing that depressed people are more likely to smoke (6, 7, 19, 21–23) and to take other substances (7, 23). Most probably, the direction of the relationship is reciprocal. Thus, whereas depression increases the likelihood of smoking, depression can also reduce the probability of short-and long-term smoking cessation (7). While researchers have emphasized that smoking should be an important target for prevention because it is so common among patients with depression (15, 22, 25), there is still little evidence whether exercise training has a positive effect on smoking among people with depression (7).

From a practical point of view, different strategies seem promising to improve cardiovascular health among patients with depression. Recommended measures include intensified screening for cardiovascular risk factors. However, it is currently not well-known whether existing decision-making tools and screening models (e.g., Cox-Framingham model) work well in people with mental illnesses (74), particularly as people with mental illnesses often are younger, have abnormal (both higher and lower) blood pressure (55, 56, 75) and are more likely to smoke than the general population (25). It is therefore recommended to use specific risk prediction models for people with mental illnesses to establish better suited thresholds for offering CVD interventions (74). It has also been emphasized that improvements in health outcomes for people with mental illnesses seem unlikely if no system-wide efforts to achieving equality in health service delivery and access are undertaken (16). Possible solutions to address systemic barriers to healthcare provision could include integrated care models such as cooperation between and co-location of physical and mental health services, the use of case managers or other liaison staff to undertake a coordination role between services, or facilitated sharing of electronic health records between physical and mental health care systems (22, 25). Another strategic approach is the promotion of lifestyle interventions targeted towards promoting healthy diet or increasing lifestyle physical activity (18, 19). Maintaining a healthy body weight through a healthy diet and regular physical activity is a key component of lowering CVD risk (25). Exercise interventions should be given high priority in clinical practice, as they are not only beneficial for cardiovascular health outcomes, but also improve patients’ mental health and cognitive functioning (7, 32, 76). For instance, low fitness proved to be more closely associated with depression than fatness (77). Accordingly, it seems important to raise awareness in psychiatrists that low CRF might be a more important predictor of morbidity and mortality than overweight and obesity (62). Thus, while reducing body weight is challenging (particularly in people with mental illnesses), improvements in CRF are achievable in relatively short time (73). In line with this notion, exercise training is included as a treatment in the context of some clinical guidelines for depression (78–82). However, given that pleasant and positive feelings during exercise have an important impact on adherence (7), it is important that exercise for individuals with depression is delivered by professionals with specific experiences in mental health care (76, 83).

The strengths of the present study were that all patients were diagnosed with depression via structured clinical interview by a psychiatrist. Although structured clinical interviews are the only well-validated method to establish a clinical diagnosis of depression, in many studies, researchers used self-report questionnaires to assess depressive symptoms (12). Additionally, detailed information was collected about participants’ use of antidepressant medication, and intake was considered as potential covariate. However, the impact on antidepressants on cardiovascular risk is not entirely clear. For instance, while tricyclic anti-depressants have been shown to increase CVD risk, some uncontrolled studies found that SSRIs (serotonin reuptake inhibitors) may reduce cardiac risk (6). Randomised placebo-controlled trials, however, failed to replicate these findings (6). Another advantage was that we not only focused on differences between patients and controls, but also examined differences within the patient sample. This seemed important as some studies showed that already mild symptoms of depression might be associated with increased cardiac risk (12). Furthermore, we controlled for a wide range of potential covariates because the association between depression and CVDs is complex and influenced by various sociodemographic factors (7) and because patients and controls differed in several sociodemographic factors. Despite these strengths, some aspects need to be considered that might limit the generalizability of our findings. First, although we controlled for antidepressant intake, we were not able to control for specific type and dose of antidepressants as there was a high heterogeneity and as some patients were taking more than one antidepressant drug (84, 85). For the same reason, it was not possible to control for pre-existing diseases although data was systematically collected in the present study. As mentioned previously, the cross-sectional analysis of the data does not allow a causal interpretation of between-group differences. Furthermore, as the present study was done in an in-patient setting, findings cannot be generalized to outpatients. Finally, it should be noted that baseline data assessment took place 2–3 weeks after admission to the hospital. Within these first weeks, depression symptom severity has decreased already substantially in the in-patient sample. Thus, improvements in symptom severity may have happened at a faster pace than changes in some of the cardiovascular risk factors.

## 5. Conclusion

Patients with depression and healthy controls differ in several cardiovascular risk markers, putting patients at increased risk for CVDs. In contrast, people with good CRF show more favourable scores across all cardiovascular risk indicators, a relationship which was observed in both healthy controls and patients with depression. Physical health of psychiatric patients should receive the clinical attention that it deserves. Lifestyle interventions targeting healthy diet and/or physical activity are recommended as a physically active and healthy lifestyle contributes equally to patients’ mental well-being and cardiovascular health.

## Data availability statement

The raw data supporting the conclusions of this article will be made available by the authors, without undue reservation.

## Ethics statement

The studies involving human participants were reviewed and approved by Ethikkommission Nordwest-und Zentralschweiz. The patients/participants provided their written informed consent to participate in this study.

## Author contributions

JB, MH, CI, UL, SM, TM, AO, and NS-K supported the patient screening and recruitment processes on the four study sites. RC and J-NK recruited the participants and collected the data. RC, JB, SB, LD, AE, MH, CI, J-NK, UL, SL, SM, TM, AO, NS-K, LZ, and OF offered thematic support. MG was responsible for conceptualizing the manuscript, conducted the statistical analyses, and wrote the first draft of the manuscript. RC carried out the final language check of the manuscript. All authors contributed to the article and approved the submitted version.

## Funding

This work was supported by the Swiss National Science Foundation (grant number 321003B-179353).

## Conflict of interest

The authors declare that the research was conducted in the absence of any commercial or financial relationships that could be construed as a potential conflict of interest.

## Publisher’s note

All claims expressed in this article are solely those of the authors and do not necessarily represent those of their affiliated organizations, or those of the publisher, the editors and the reviewers. Any product that may be evaluated in this article, or claim that may be made by its manufacturer, is not guaranteed or endorsed by the publisher.
